# NATE: Non-pArameTric approach for Explainable credit scoring on imbalanced class

**DOI:** 10.1371/journal.pone.0316454

**Published:** 2024-12-31

**Authors:** Seongil Han, Haemin Jung

**Affiliations:** 1 School of Computing & Mathematical Sciences, University of London, Birkbeck College, London, United Kingdom; 2 Department of Industrial & Management Engineering, Korea National University of Transportation, Chungju, South Korea; National Institute of Electronics and Information Technology, INDIA

## Abstract

Credit scoring models play a crucial role for financial institutions in evaluating borrower risk and sustaining profitability. Logistic regression is widely used in credit scoring due to its robustness, interpretability, and computational efficiency; however, its predictive power decreases when applied to complex or non-linear datasets, resulting in reduced accuracy. In contrast, tree-based machine learning models often provide enhanced predictive performance but struggle with interpretability. Furthermore, imbalanced class distributions, which are prevalent in credit scoring, can adversely impact model accuracy and robustness, as the majority class tends to dominate. Despite these challenges, research that comprehensively addresses both the predictive performance and explainability aspects within the credit scoring domain remains limited. This paper introduces the Non-pArameTric oversampling approach for Explainable credit scoring (NATE), a framework designed to address these challenges by combining oversampling techniques with tree-based classifiers to enhance model performance and interpretability. NATE incorporates class balancing methods to mitigate the impact of imbalanced data distributions and integrates interpretability features to elucidate the model’s decision-making process. Experimental results show that NATE substantially outperforms traditional logistic regression in credit risk classification, with improvements of 19.33% in AUC, 71.56% in MCC, and 85.33% in F1 Score. Oversampling approaches, particularly when used with gradient boosting, demonstrated superior effectiveness compared to undersampling, achieving optimal metrics of AUC: 0.9649, MCC: 0.8104, and F1 Score: 0.9072. Moreover, NATE enhances interpretability by providing detailed insights into feature contributions, aiding in understanding individual predictions. These findings highlight NATE’s capability in managing class imbalance, improving predictive performance, and enhancing model interpretability, demonstrating its potential as a reliable and transparent tool for credit scoring applications.

## Introduction

Credit risk refers to the potential loss arising from an applicant’s creditworthiness [1]. This form of risk can significantly affect non-performing financial obligations, which are closely associated with bankruptcy. Given its considerable impact on the financial sustainability of institutions, accurately assessing and managing credit risk through precise credit scoring is essential [2].

Logistic Regression (LR) is considered the industry standard for credit scoring and is supported by research literature for its acceptable performance and interpretability compared to other classifiers [3, 4]. However, Logistic Regression (LR) exhibits limited classification performance on non-linear credit scoring datasets due to its inability to capture non-linear relationships. In contrast, non-parametric tree-based models can detect non-linear relationships between credit risk features and creditworthiness that LR fails to identify, by exploring the relationships within partitioned samples [5].

Numerous studies have demonstrated that non-parametric tree-based ensemble models outperform single algorithms, such as LR, in credit scoring applications [6–8]. Consequently, these ensemble approaches have garnered significant attention and are now considered mainstream in the field of credit scoring [4].

However, non-parametric models, such as tree-based ensemble algorithms, are challenging to interpret despite their high predictive power. Black box Artificial Intelligence (AI) models, which are not easily explainable, are unsuitable for the finance sector [9]. In contrast, parametric models like LR offer high interpretability, albeit with limited predictive performance.

Recently, considerable efforts have been dedicated to applying advanced Machine Learning (ML) technologies to the field of credit scoring. However, two primary challenges remain: the need for model interpretability and the imbalanced class distribution within datasets [2]. When the disparity between class instances is minimal, machine learning models tend to maintain prediction accuracy. However, significant class imbalance complicates the learning process, often leading models to favor the majority class, which impacts accuracy [10]. Compared to balanced datasets, imbalanced datasets present substantial challenges for classification models and often result in misclassification, particularly when assessing the creditworthiness of loan applicants.

In real-world credit scoring, datasets are frequently imbalanced due to the predominance of applicants with good credit compared to those with poor credit. This notable disparity, known as class imbalance, is a characteristic of Low Default Portfolios (LDPs) in credit scoring, where the majority of loan applicants have good credit, while only a small minority are considered high risk. Consequently, these datasets display pronounced class imbalances, with significantly fewer observations in the minority class (bad credit applicants) compared to the majority class (good credit applicants) [3].

To address class imbalance, various re-sampling methods have been proposed [11]. Among these, the Synthetic Minority Oversampling TEchnique (SMOTE) is one of the most widely employed for augmenting minority class samples. While oversampling techniques like SMOTE utilize all available information, undersampling approaches often result in a loss of valuable data [12].

In response to the trade-off between explainability and predictive performance within credit scoring, we propose integrating non-parametric tree-based models with ‘TreeExplainer’. These interpretable models enable comprehensive analysis of model predictions using SHAP (SHapley Additive exPlanations) values, without sacrificing predictive accuracy. SHAP, introduced by Lundberg and Lee [13], elucidates model predictions by evaluating the contribution of each feature at both global and local levels.

This study aims to introduce Non-pArameTric tree-based ensemble models for Explainable credit scoring, named NATE. The NATE framework integrates models such as Random Forest (RF), Gradient Boosting (GB), and eXtreme Gradient Boosting (XGB) to enhance classification performance and improve interpretability of model predictions in the context of imbalanced credit scoring datasets.

To thoroughly assess model performance, the dataset size will be adjusted using various sampling methods, allowing for an examination of how these ensemble models respond to different levels of class imbalance. This analysis places particular emphasis on evaluating the robustness of non-parametric tree-based models. Furthermore, the effects of undersampling and oversampling techniques on classification performance for credit scoring will be evaluated across a range of class imbalances. Finally, predictions will be interpreted using eXplainable Artificial Intelligence (XAI) methods to offer a transparent understanding of the classification process within credit scoring.

The key contributions of this study are as follows:

- To demonstrate the efficacy of non-parametric models on non-linear datasets for credit scoring: This study aims to showcase the superior performance of non-parametric models when applied to non-linear datasets in the context of credit scoring.
- To present the standard oversampling method by SMOTE for synthesizing the minority class on imbalanced datasets, in comparison to the undersampling method by NearMiss: This research compares the effectiveness of the SMOTE oversampling technique with the NearMiss undersampling method in addressing class imbalance in credit scoring datasets.
- To propose the architecture of non-parametric models for non-linear and imbalanced credit scoring datasets: This work introduces a novel architecture for non-parametric models designed to handle the complexities of non-linear and imbalanced credit scoring datasets.
- To achieve the explainability aspect for practical application in credit scoring through XAI, alongside high predictive performance of the proposed non-parametric models: This study emphasizes the importance of explainability in practical credit scoring applications by leveraging eXplainable Artificial Intelligence (XAI) techniques, while also ensuring that the proposed non-parametric models deliver high predictive accuracy.

We hypothesize that the proposed NATE models will not only enhance classification performance by capturing non-linearity in imbalanced datasets but also develop models that are explainable, providing clear reasons for credit scoring predictions.

The remainder of this paper is organized as follows: Section II reviews related studies. Section III outlines the proposed NATE model and its underlying concepts. Section IV presents the experimental results and evaluates the performance of NATE, comparing it with parametric models using both oversampling and undersampling techniques. Section V discusses the key findings, limitations, and potential directions for future work. Finally, Section VI concludes with a summary of the study’s findings.

## Related work

This section reviews related studies on the development of explainable models for interpretability and hybrid and ensemble approaches for improved predictive performance.

### Explainability as eXplainable AI in credit scoring

Explainability is essential in the field of credit scoring for both financial institutions and credit applicants. Chen *et al.* (2016) [14] articulated that Linear Discriminant Analysis (LDA) and Logistic Regression (LR) are capable of identifying the optimal linear combination of input features, thereby enhancing the interpretability of credit scoring models. Nonetheless, despite this advantage, statistical models frequently suffer from limited predictive power, which is regarded as their principal weakness [5].

Tree-based machine learning ensemble classifiers, such as Random Forest (RF) and Gradient Boosting (GB), are among the most widely used non-linear predictive models [15, 16]. These models are employed in domains where it is imperative that predictions are both accurate and explainable, such as in medicine, pharmacology, and finance [17]. In these fields, achieving a balance between accuracy and explainability is essential. Explainability refers to the ability to understand how machine learning classifiers utilize input features to make predictions [16].

As an aspect of model interpretability, Logistic Regression (LR) utilizes the logistic function, allowing for straightforward interpretation of its coefficients. However, this approach overlooks interactions between variables due to its reliance on a linear decision boundary. Conversely, tree-based algorithms such as Random Forest (RF) and Gradient Boosting (GB) are adept at training on complex and non-linear decision boundaries, making the interpretation of their predictions more challenging [18]. While Decision Trees (DT) can be interpreted by examining their decision paths, the use of multiple trees in tree-based ensemble models reduces the interpretability of the predictions.

Recently, substantial research efforts have been dedicated to the field of eXplainable AI (XAI). As noted above, the importance of both accuracy and interpretability has driven extensive studies in fields such as medicine, pharmacology, biology, and finance. Lundberg *et al.* (2020) [16] proposed a method to make tree-based models interpretable by assessing input contributions. In an earlier study, Lundberg and Lee [13] introduced ‘TreeExplainer’ with SHAP (SHapley Additive exPlanations), a unified approach grounded in Shapley values from coalitional game theory. Shapley (1953) [19] introduced these values, which represent the average contribution of each player in a cooperative game using the concept of pay-off. These Shapley values can be applied to estimate the contribution of each input feature to the predictions of machine learning models [9]. This approach allows for the explanation of machine learning model predictions, facilitating the analysis and understanding of how each feature contributes to the target feature globally and how specific samples are predicted locally by the SHAP values of their features. Furthermore, Lundberg and Lee (2017) [13] also proposed ‘LinearExplainer,’ which enables global and local analysis of Logistic Regression (LR) predictions using the same principles, despite the inherent interpretability of LR coefficients.

A range of model-agnostic XAI techniques, including SHAP [16] and LIME (Local Interpretable Model-agnostic Explanations) [20], offer substantial potential for improving the interpretability of machine learning models [2].

Gramegna and Giudici (2021) [21] assessed the discriminative power of two prominent XAI techniques, SHAP and LIME, in the context of credit risk evaluation. Their findings suggest that these XAI models could serve as a foundation for post-processing feature extraction in credit risk applications. Hjelkrem and Lange (2023) [22] proposed two deep learning credit scoring models utilizing customer transaction descriptions. Their study assessed the predictive performance of these models and employed SHAP for interpretability, offering insights at a global level and clarifying specific application rejections. Talaat *et al.* (2024) [23] proposed a model for predicting credit card defaults by integrating deep learning with XAI techniques, utilizing SHAP to enhance interpretability in credit risk assessment. This approach not only achieves competitive predictive accuracy but also provides valuable insights into key factors influencing credit card default risk. The study contributes to the field of XAI in finance by offering a balanced solution that combines predictive accuracy with transparency, advancing the development of interpretable and trustworthy credit scoring models.

On the other hand, feature importance measures based on information gain or Gini impurity in tree-based ensemble models can assess the significance of each input feature and offer insights into the underlying reasons for predictions. However, this approach has limitations, as it reflects feature importance across the entire dataset but does not elucidate each feature’s contribution to individual predictions.

### Hybrid and ensemble approaches for improved predictive performance

Hybrid and ensemble approaches have been utilized in medicine and pharmacology to improve predictive performance over existing models.

Raza *et al.* (2024) [24] introduced the AIPs-DeepEnC-GA (Anti-Inflammatory Peptides-Deep Ensemble Classifier with Genetic Algorithm), an advanced genetic algorithm-based deep learning approach designed for the prediction of anti-inflammatory peptides. This model employs a hybrid feature integration strategy that incorporates embedded sequential feature integration to enhance prediction accuracy. The minimum Redundancy Maximum Relevance (mRMR) method is applied to select the optimal features from the combined feature set. Across all datasets utilized in the study, the AIPs-DeepEnC-GA model outperforms existing computational models, demonstrating superior predictive capability. Rukh *et al.* (2024) [25] proposed the StackedEnC-AOP (Stacked Ensemble Classifier for AntiOxidant protein Prediction), an advanced approach for classifying antioxidant proteins. This model enhances predictive accuracy by integrating Discrete Wavelet Transform (DWT) with matrix encoding and incorporating additional physiochemical descriptors. Key features are optimized through mRMR and trained within a stacking-based ensemble meta-model to improve computational efficiency. The StackedEnC-AOP model achieved superior performance compared to existing methods. Ullah *et al.* (2024) [26] introduced DeepAVP-TPPred (Deep Antiviral Peptide-Transformed Image-Based Localized Descriptors and Binary Tree Growth Algorithm), a machine learning model for predicting antiviral peptides. This model integrates custom image-based feature sets and information-based features, optimized through a binary tree growth algorithm, to create an efficient feature set for training a deep neural network. The DeepAVP-TPPred achieved superior accuracy and generalization compared to existing models. Akbar *et al.* (2024) [27] introduced the iAFPs-Mv-BiTCN (integrated AntiFungal Peptides-Multi-view Bidirectional Temporal Convolutional Network), a computational approach utilizing Bidirectional Temporal Convolutional Networks for the prediction of antifungal peptides. This model employs a transform matrix, self-attention transformer, and fastText-based word embedding to effectively represent peptide samples in numerical form. SHAP interpolation-based feature selection is applied to identify optimal features from the hybrid vector. The iAFPs-Mv-BiTCN model achieved enhanced predictive accuracy over existing computational models. Raza *et al.* (2023) [28] introduced AIPs-SnTCN (Anti-Inflammatory Peptides-Self-normalized Temporal Convolutional Network), an advanced model for predicting anti-inflammatory peptides, leveraging word embedding methods like skip-gram and attention-based Bidirectional Encoder RepresenTations (BERT) along with Conjoint Triad Features (CTF) to capture structural information. A fused vector of word embedding and sequential features enhances encoding, and Support Vector Machine-based Recursive Feature Elimination (SVM-RFE) is applied for optimal feature selection. AIPs-SnTCN, which was trained using a Self-normalized Temporal Convolutional Network (SnTCN), outperformed existing models. Akbar *et al.* (2024) [29] developed the Deepstacked-AVPs model to improve the classification accuracy of AntiViral Peptides (AVPs). The model integrates a tri-segmentation-based Position-Specific Scoring Matrix (PSSM-TS), word2vec-based semantic features, and Composition/Transition/Distribution-Transition (CTDT) descriptors to capture structural and physiochemical properties. Optimal features are selected and trained within a stacked-ensemble classifier, resulting in superior predictive performance compared to existing models.

These studies highlight their significant potential for applications in fields such as medicine and biology, with particular relevance to drug development, peptide-based therapeutic design, and pharmaceutical science.

Furthermore, numerous studies have attempted to optimize classification performance in the context of imbalanced classes in credit scoring datasets. One approach to addressing class imbalance is through resampling techniques, as previously discussed. These techniques aim to adjust the number of samples and balance the classes in the original data by either reducing the number of majority class instances or increasing the number of minority class instances. Given that oversampling techniques utilize all available information, they have been extensively preferred over undersampling methods in research.

Chawla *et al.* (2002) [30] introduced the Synthetic Minority Oversampling TEchnique (SMOTE). This method generates new samples between instances of the minority class and their neighbors within the same class using local information from the K-Nearest Neighbors (KNN) algorithm, rather than simply duplicating the minority class samples [4]. Han *et al.* (2005) [31] proposed Borderline-SMOTE (B-SMOTE), and Hu *et al.* (2009) [32] introduced Modified-SMOTE (M-SMOTE). Both B-SMOTE and M-SMOTE are variants of SMOTE designed to overcome its limitations and improve classification performance, recognizing that minority class samples and their neighboring samples might belong to different classes when SMOTE oversamples the minority class. He *et al.* (2008) [33] proposed the ADAptive SYNthetic (ADASYN) sampling approach for imbalanced datasets, which uses a weighted distribution in the samples of the minority class. This method aims to mitigate biased performance caused by class imbalance. Batista *et al.* (2004) [34] conducted comparative experiments employing resampling methods, such as oversampling and undersampling, on various imbalanced datasets. Their findings indicated that oversampling methods generally produce more accurate results than undersampling methods in terms of AUROC. Brown and Mues (2012) [3] provided comparative analyses using multiple algorithms to evaluate the impact of resampling methods on imbalanced credit scoring datasets for each classifier. The results revealed that Random Forest (RF) and Gradient Boosting (GB) performed better than Logistic Regression (LR). Marques *et al.* (2013) [35] explored resampling techniques on imbalanced credit scoring datasets and demonstrated that resampling methods, particularly oversampling approaches, consistently enhance classification performance. Zieba *et al.* (2016) [36] employed eXtreme Gradient Boosting (XGB) for bankruptcy prediction on credit datasets, showing superior results compared to benchmarks. Xiao *et al.* (2016) [8] proposed an ensemble approach based on supervised clustering to partition samples of each class, thereby improving classification performance in credit scoring. Xia *et al.* (2017) [7] utilized the XGB model with Bayesian hyperparameter tuning, showcasing both improved interpretability and enhanced classification performance in credit scoring.

These studies have demonstrated that the use of non-parametric tree-based ensemble approaches has been increasing to enhance the performance of credit scoring. Consequently, non-parametric tree-based ensemble models, when combined with ‘TreeExplainer,’ can overcome the predictive performance limitations of Logistic Regression (LR), the industry standard, while also adding interpretability to predictions in the domain of credit scoring.

Furthermore, tree-based ensemble models are more suitable for capturing non-linearity in datasets. According to experimental results on medical datasets by Lundberg *et al.*, (2020) [16], an increase in dataset non-linearity correlates with higher explanation and accuracy errors, despite the stability of tree-based Gradient Boosting (GB). This indicates that both explainability and accuracy decline as non-linearity increases, due to the inclusion of irrelevant features in the model and the diminishing clarity of the relationship between the target feature and training features [16]. This suggests that tree-based models are preferable to linear models when they achieve comparable accuracy in each case.

## Methods

### Overview

The Non-pArameTric approach for Explainable credit scoring on imbalanced class (NATE) methodology comprises four sequential stages:

1. Collect the GMSC (Give Me Some Credit) dataset (https://kaggle.com/competitions/GiveMeSomeCredit)
2. Balance the dataset by either undersampling the majority class (good credit samples) using NearMiss or oversampling the minority class (bad credit samples) using SMOTE
3. Perform classification predictions using both parametric and non-parametric models on the oversampled and undersampled datasets for comparative analysis
4. Explain the models using TreeExplainer within the framework of XAI

These stages must be processed sequentially to achieve the required levels of effectiveness.

The GMSC dataset is employed to evaluate the performance of tree-based ensemble classifiers under varying imbalance ratios, in comparison to Logistic Regression (LR). This dataset contains demographic information, payment behavior, and delinquency data for the samples and is derived from the Kaggle competition “Give Me Some Credit”. It is widely recognized as a benchmark in credit scoring research [18].

The dataset comprises 150,000 samples, with approximately 140,000 non-defaulted credit samples and 10,000 defaulted credit samples. Bad credit samples are identified when the target feature, ‘SeriousDlqin2yrs’, is specified as 1, indicating that the applicant has defaulted on the loan. Conversely, good credit samples are classified as those where the label is specified as 0, meaning the applicant has fulfilled their financial obligation. This classification results in a binary class label. The dataset was selected for its non-linearity in the results [18], to validate the robustness of non-parametric models in comparison to parametric models.

**Fig 1:**
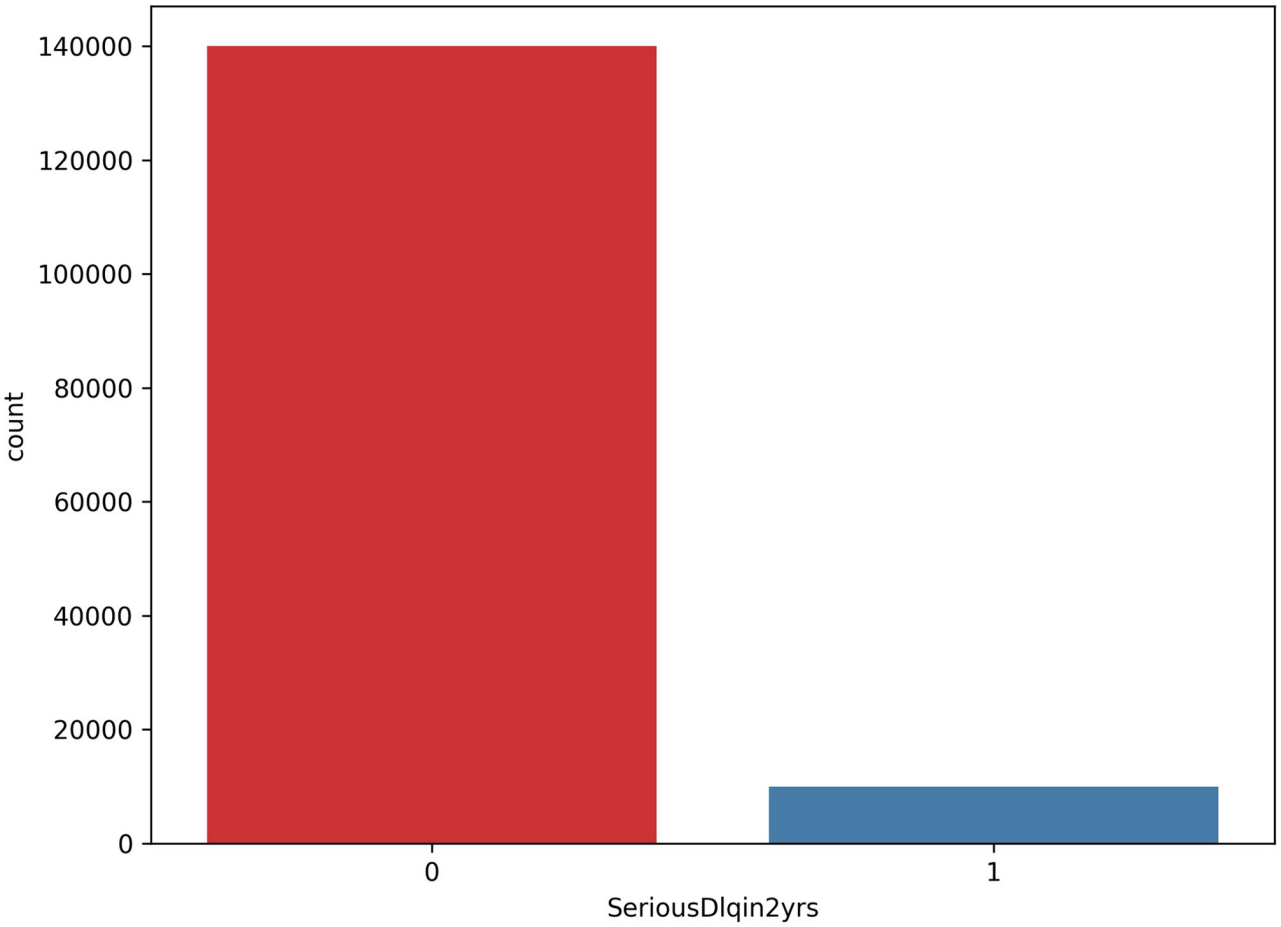
Class imbalance on GMSC dataset. ‘0’ means not defaulted (good credit) and ‘1’ means defaulted (bad credit).

The initial dataset reveals that the minority class constitutes 6.684% of the total, with an Imbalance Ratio (IR) of 13.961, calculated as the ratio of majority class instances to minority class instances. The dataset comprises 10 features, excluding the target feature, all of which can be directly interpreted within a credit scoring system. Tables 1 and 2 provides a detailed description of the dataset, while Fig 1 illustrates the class imbalance present in the dataset.

**Table 1:** GMSC dataset (IR: Imbalance Ratio).

Dataset	#Samples	#Good	#Bad	IR	#Features
GMSC	150,000	139,974	10,026	13.961	10

**Table 2:** Features on GMSC dataset used in NATE.

Feature	Description	Type
**SeriousDlqin2yrs** *	Applicant experienced 90 days past due delinquency or worse	Y/N (1 or 0)
RevolvingUtilizationOfUnsecuredLines	Total balance on credit cards and personal lines of credit	percentage
age	Age of in years	integer
NumberOfTime30-59DaysPastDueNotWorse	Number of times applicant has been 30-59 days past due but no worse in the last 2 years.	integer
DebtRatio	Monthly debt payments, alimony,living costs divided by monthly gross income	percentage
MonthlyIncome	Monthly income	real
NumberOfOpenCreditLinesAndLoans	Number of Open loans (installment like car loan or mortgage) and Lines of credit	integer
NumberOfTimes90DaysLate	Number of times applicant has been 90 days or more past due.	integer
NumberRealEstateLoansOrLines	Number of mortgage and real estate loans including home equity lines of credit	integer
NumberOfTime60-89DaysPastDueNotWorse	Number of time applicant has been 60-89 days past due but no worse in the last 2 years.	integer
NumberOfDependents	Number of dependents in family excluding themselves (spouse, children etc.)	integer

*SeriousDlqin2yrs is a feature for class label.

### The framework of NATE

To achieve different imbalance ratios or proportions in the credit samples of the dataset, the class distribution has been adjusted. This adjustment is accomplished through sampling techniques that alter the distribution of the imbalanced dataset by either undersampling good credit samples or oversampling bad credit samples. This is necessary because the number of good credit samples significantly exceeds the number of bad credit samples, as previously described.

Two standard techniques commonly used to address class imbalance are NearMiss for undersampling and SMOTE for oversampling. Tables 3 and 4 present the resampled credit dataset, illustrating different imbalance ratios and the distributions of good credit and bad credit samples, respectively.

**Table 3:** Under-sampled dataset.

Undersampling #good	7%(original)	13%	24%	33%	50%
Imbalance ratio	13.96	6.67	3.12	2	1
#Not dafaulted	139,974	66,840	31,331	20,052	10,026
#Defaulted	10,026	10,026	10,026	10,026	10,026

**Table 4:** Over-sampled dataset.

Oversampling #bad	7%(original)	13%	24%	33%	50%
Imbalance ratio	13.96	6.67	3.12	2	1
#Not defaulted	139,974	139,974	139,974	139,974	139,974
#Defaulted	10,026	20,996	44,791	69,987	139,974

**Fig 2:**
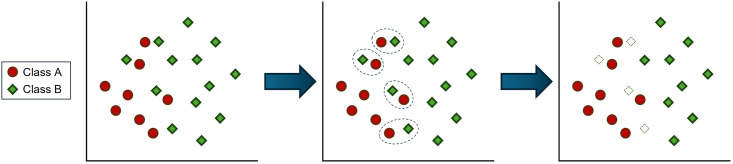
Class distribution balanced through NearMiss undersampling.

The undersampling method NearMiss, proposed by Mani and Zhang (2003) [37], has been applied to the original dataset. NearMiss is an undersampling technique that reduces the majority class samples using a distance-based or nearest-neighbor method. When majority class samples are near minority class samples, the majority class samples are eliminated until the class distributions are balanced. Fig 2 illustrates the balanced class distribution achieved through NearMiss.

From the initial 139,974 good credit samples, 66,840 samples were used to achieve a class imbalance ratio of 6.67, resulting in the bad credit class constituting 13% of the total. For a class imbalance ratio of 2, 20,052 samples were used, bringing the percentage of bad credit samples to 33%. To reach a class imbalance ratio of 1, 10,026 samples were used, resulting in the bad credit class making up 50% of the total. NearMiss effectively undersampled the majority class to match the total number of minority samples, thereby balancing the dataset.

**Fig 3:**
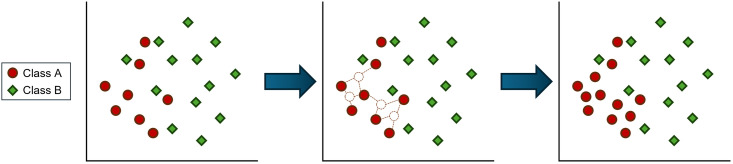
Class distribution balanced through SMOTE oversampling.

**Fig 4:**
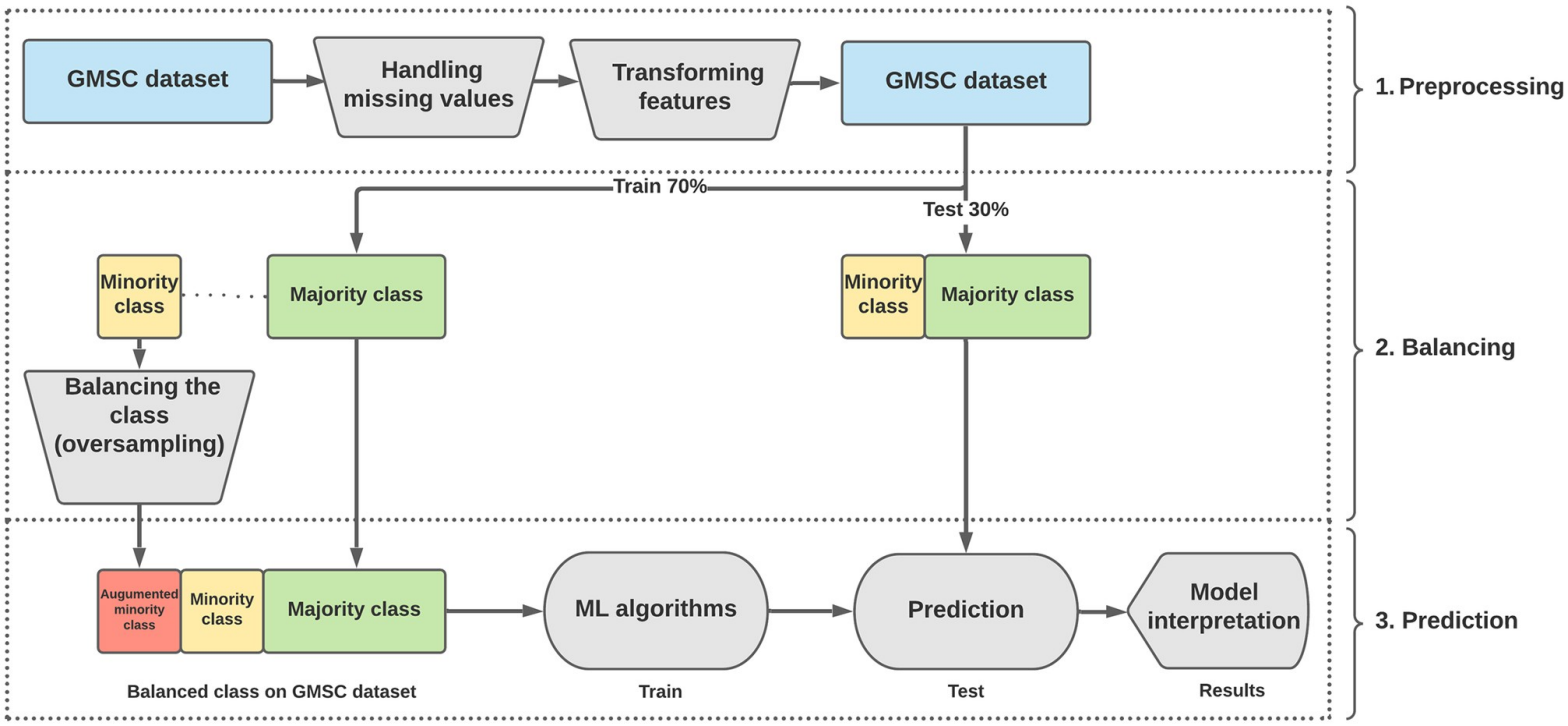
The system architecture of NATE.

Conversely, the Synthetic Minority Oversampling TEchnique (SMOTE), proposed by Chawla *et al.* (2002) [30], has been applied to the original dataset. SMOTE generates synthetic samples using local information from the k-Nearest Neighbors (KNN). After selecting a sample *x*_*i*_ from the minority class, its neighboring sample $\hat{x_{i}}$ is identified using KNN, as illustrated in Fig 3. A synthetic sample *x*_*synthetic*_ is then randomly generated between existing minority class samples using a parameter λ in the interval [0, 1], as shown in Fig 3. Finally, the number of minority class samples is increased to match the number of majority class samples, achieving balance in the dataset, as depicted in Fig 4. The SMOTE methodology can be formally expressed as follows:

$$\begin{array}{c} x_{\text{synthetic}}=x_{i}+\lambda \left(\hat{x_{i}}-x_{i}\right),\;\lambda \in \left[0,1\right] \end{array}$$ (1)

From the 10,026 bad credit samples, SMOTE was applied to achieve various class imbalance ratios: 20,996 samples were used to create an imbalance ratio of 6.67, resulting in the bad credit class constituting 13% of the total; 69,987 samples were used to achieve an imbalance ratio of 2, making up 33% of the total; and 139,974 samples were used to achieve a balance ratio of 1, where the bad credit class comprised 50% of the total. Ultimately, SMOTE oversampled the minority class to match the total number of majority class samples, thus balancing the dataset.

As a result of resampling the class distribution, 8 datasets were generated from the original dataset. The performance of non-parametric tree-based ensemble models can be evaluated and compared against the Logistic Regression (LR) model using the original dataset, which exhibits a class imbalance of 6.694% and an imbalance ratio of 13.961, serving as a benchmark.

After adjusting the class distribution through resampling techniques, the prediction for credit scoring is conducted using tree-based ensemble algorithms, employing a non-parametric approach.

Fig 4 illustrates the overall system architecture. This experiment aims to assess whether non-parametric algorithms combined with resampling techniques can enhance classification performance in datasets with class imbalance, in comparison to the standard Logistic Regression (LR) method applied to the original imbalanced dataset.

ML classifiers including tree-based models are employed for performance comparison and they are as follows:

Logistic Regression (LR) has been widely utilized in the field of credit scoring due to its straightforward interpretability [38]. Linear Discriminant Analysis (LDA) represents a statistical learning technique designed to model a linear combination of features that effectively discriminates between classes [39]. Both LR and LDA are classified as parametric models. In contrast, the K-Nearest Neighbour (KNN) algorithm functions as a distance-based classifier, determining the distance between input feature vectors and assigning points to the class of their K-nearest neighbours [40]. KNN is recognized as a non-parametric algorithm. Decision Trees (DT) operate by recursively partitioning the dataset based on specific information criteria to achieve classification [41], and are also non-parametric models. Naive Bayes (NB) is a probabilistic classifier grounded in Bayesian theorem [42], generally considered a parametric model, though it can be either parametric or non-parametric depending on the parameter configuration. Random Forest (RF) is an ensemble method that integrates multiple decision tree classifiers to enhance predictive performance [43]. Gradient Boosting (GB) represents a boosting technique that amalgamates weak classifiers into a robust model, improving classification accuracy [44]. In this study, decision trees serve as the base learners in the GB experiment. Both RF and GB are non-parametric models. Extreme Gradient Boosting (XGB) is a non-parametric model that builds upon the tree models of GB to classify diverse tasks and optimize performance [45].

The primary performance metric used is the Area Under the Receiver Operating Characteristic Curve (AUROC). Given that classification accuracy in imbalanced datasets is often skewed towards the majority class, AUROC is recognized as the standard metric for evaluating classification performance in the context of imbalanced datasets [10, 46]. Moreover, to further ensure robustness and generalizability of the results, Matthews Correlation Coefficient (MCC) and F1 score were also employed as performance metrics. AUROC, along with MCC and F1 score, collectively provides a comprehensive evaluation of classification performance, particularly in the context of imbalanced datasets.

Following the prediction by machine learning models, the results can be interpreted using TreeExplainer and LinearExplainer for model explanation.

As previously discussed, SHAP, as proposed by Lundberg and Lee (2017) [13], facilitates the explanation of individual predictions by estimating the contribution of each feature. TreeExplainer is used for interpreting tree-based models, while LinearExplainer is employed for interpreting logistic regression models. SHAP representations are characterized by an additive feature attribution framework utilizing Shapley values [19], which can be expressed as follows:

$$\begin{array}{c} g\left(z^{\prime }\right)=\phi_{0}+\sum_{i=1}^{M}\phi_{i}z_{i}^{\prime } \end{array}$$ (2)

Where g represents the model for explanation, specifically the approximation of the prediction, *z*′ ∈ {0, 1}^*M*^ denotes the coalitional vector (also referred to as “simplified features” in Lundberg and Lee’s study [13]), with 1 indicating the feature is ‘present’ and 0 indicating it is ‘absent’. *M* is the maximum coalitional size, corresponding to the number of input features utilized, and $\phi_{i}\in R$ represents the attribution for feature *i*. For instance, if the values for all features are present (*z*′ = 1), then Eq 2 can be simplified as follows:

$$\begin{array}{c} g\left(x^{\prime }\right)=\phi_{0}+\sum_{i=1}^{M}\phi_{i} \end{array}$$ (3)

SHAP adheres to the properties of local accuracy, missingness, and consistency. These characteristics were demonstrated by Lundberg and Lee (2017) [13]. Their proof reconciled the differences between SHAP and Shapley values for interpreting machine learning models. To bridge this gap and facilitate the interpretation of machine learning models, the Shapley value is defined as follows:

$$\begin{array}{c} \phi_{i}\left(f,x\right)=\frac{1}{|M|!}\sum_{z^{\prime }\subseteq x^{\prime }}|z^{\prime }\left|!(M-\right|z^{\prime }\left|-1\right)![f_{x}\left(z^{\prime }\right)-f_{x}\left(z^{\prime }\backslash j\right)] \end{array}$$ (4)

Where *f* denotes the trained model, *z*′\*j* indicates that $z_{j}^{\prime }=0$, *x* represents the input features, *x*′ denotes the M selected input features, and *f*_*x*_(*z*′) − *f*_*x*_(*z*′\*j*) represents the feature contribution of sample *i* for each prediction [9].

As previously discussed, the characteristics of Shapley values can be applied to the interpretation of models as follows [9]:

- Local accuracy: Shapley values provide quantifiable measures by constructing an explainable model that estimates the original model in an additive form locally for a specific sample *x*.
- Missingness: If a feature is absent (i.e., a feature value is 0), the Shapley value for that feature is 0, indicating that a missing feature has zero attribution.
- Consistency: If the contribution of a feature increases or decreases, regardless of other features in the model, the corresponding Shapley value will also increase or decrease accordingly.

Utilizing these characteristics, SHAP calculates the Shapley values of the features to explain predictions both locally and globally in credit scoring models [16].

As illustrated in Fig 4, the overall system architecture for the proposed study can be summarized as follows:

Firstly, the credit scoring dataset is pre-processed. In this stage, features undergo transformation using methods such as standardization and normalization, and missing values are addressed accordingly.

Secondly, feature extraction techniques, including feature engineering, are applied to the datasets. This process involves calculating feature importance, optimizing feature subsets, selecting model-based features, and resampling imbalanced datasets to achieve a specific ratio or balanced class distribution. The goal is to identify the most effective and least redundant features or subsets prior to training machine learning models.

Ensemble classifiers such as Random Forest (RF), Gradient Boosting (GB), and eXtreme Gradient Boosting (XGB) are then employed to train the model and perform classification on datasets with different imbalance ratios that have been resampled using various sampling techniques. This approach aims to validate the impact of class imbalance and imbalance ratios on the performance of tree-based ensemble classifiers in the domain of credit scoring.

The performance outcomes of non-parametric models, including Random Forest (RF), Gradient Boosting (GB), and eXtreme Gradient Boosting (XGB), are compared to the performance results of parametric models such as Logistic Regression (LR), which serves as a benchmark and is the most frequently used classifier in the domain of credit scoring, as previously discussed.

Finally, interpretation using ‘Explainer’ is conducted to understand the contributions of input features to the prediction of credit scores, both at a local and global level.

## Results

This section evaluates classification performance through resampling techniques and interprets the predictions using the ‘Explainer’.

**Fig 5:**
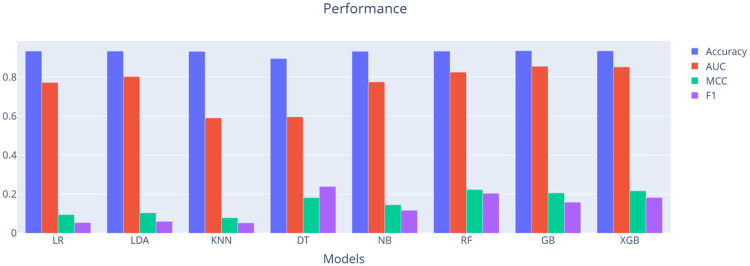
Performance comparison of machine learning models across accuracy, AUC, MCC, and F1 score on GMSC dataset.

Table 5 and Fig 5 present a comparative analysis of accuracy, AUC, MCC and F1 score for both parametric and non-parametric classifiers applied to the original imbalanced credit scoring dataset. Performance metrics, including accuracy, AUC, MCC and F1 score, were computed using 5-fold cross-validation. Due to the substantial disparity in class sample sizes, resulting in a significantly imbalanced dataset, the accuracy metric exhibits bias towards the majority class, as evidenced in Table 5 and Fig 5. This observation indicates that accuracy is an inadequate measure for performance evaluation in this scenario. Therefore, AUC, MCC and F1 score are utilized as a more reliable performance metric.

**Table 5:** Performance comparison of machine learning models across accuracy, AUC, MCC, and F1 score on GMSC dataset.

Classifier	Accuracy	AUC	MCC	F1
LR	0.9323	0.7716	0.0948	0.0539
LDA	0.9325	0.8018	0.1042	0.0599
KNN	0.9310	0.5901	0.0787	0.0525
DT	0.8941	0.5955	0.1819	**0.2386**
NB	0.9311	0.7749	0.1451	0.1170
RF	0.9322	0.8242	**0.2229**	0.2037
GB	**0.9343**	**0.8542**	0.2058	0.1579
XGB	0.9335	0.8515	0.2165	0.1827
Bayes Net [47]	0.9450	0.8550	N/A	N/A

### Benchmarking results on original dataset

As shown in Table 5 and Fig 5, Gradient Boosting (GB) achieved the highest accuracy and AUC, while Random Forest (RF) yielded the highest MCC, and Decision Tree (DT) demonstrated the highest F1 score. When compared to parametric algorithms such as Logistic Regression (LR), Linear Discriminant Analysis (LDA), and Naive Bayes (NB), non-parametric ensemble classifiers such as Random Forest (RF), GB, and eXtreme Gradient Boosting (XGB) demonstrate superior performance in the accuracy, AUC, MCC and F1 score, consistent with previous studies. It has been established that non-parametric models like RF, GB, and XGB possess the capability to effectively capture local features in imbalanced and non-linear datasets [3]. Consequently, the exploration of non-parametric models in terms of classification performance warrants further experiments, based on the justification provided by these results.

### Performance comparison on resampled dataset

The performance of classifiers is evaluated across different class ratios using 9 datasets, including the original dataset, to validate the impact of class imbalance.

To conduct a robust comparative analysis between parametric and non-parametric models, 5-fold cross-validation was utilized. Model performance was evaluated using AUC, MCC, and F1 score metrics to ensure a reliable assessment. At the data level, these metrics, applied with 5-fold cross-validation, were intended to enhance the model’s generalizability across the entire GMSC dataset. This iterative training and validation approach enabled a comprehensive evaluation of model robustness. Given that tree-based models are prone to overfitting without pruning, pruning techniques were incorporated into the NATE framework to reduce unnecessary exploration depth and thereby mitigate overfitting risks.

Furthermore, grid search with 5-fold cross-validation was used for hyperparameter tuning, optimizing model complexity to further prevent overfitting and improve generalization performance. This optimization process was carried out on imbalanced datasets (with imbalance ratios of IR = 6.67, IR = 3.12, and IR = 2) as well as a balanced dataset (IR = 1) to ensure optimal performance for both SMOTE and NearMiss resampling techniques. The details of the hyperparameter search space are provided in Table 6. These combined strategies allowed the NATE framework to achieve a balance between model accuracy and robustness across diverse data contexts while mitigating the risk of overfitting.

**Table 6:** Searching space for hyperparameters in Table 7.

Algorithm	Hyperparameter	Value
LR	penalty	{‘none’, ‘L1’, ‘L2’, ‘elasticnet’}
	solver	{‘newton-cg’, ‘lbfgs’, ‘liblinear’}
RF	n_estimators	[100, 1000]
	max_features	{‘auto’, ‘sqrt’}
	max_depth	[1, 20]
	min_samples_split	{2, 5, 10}
	min_samples_leaf	{1, 2, 4, 8}
	bootstrap	{‘True’, ‘False’}
GB	n_estimators	[100, 1000]
	learning_rate	{‘0.01’, ‘0.1’}
	max_depth	[1, 20]
	min_samples_split	{2, 5, 10}
	min_samples_leaf	{1, 2, 4, 8}
	subsample	{0.8, 1.0}
XGB	n_estimators	[100, 1000]
	learning_rate	{‘0.01’, ‘0.1’}
	max_depth	[1, 20]
	min_child_weight	{2, 5, 10}
	subsample	{0.8, 1.0}
	colsample_bytree	{0.8, 1.0}

**Fig 6:**
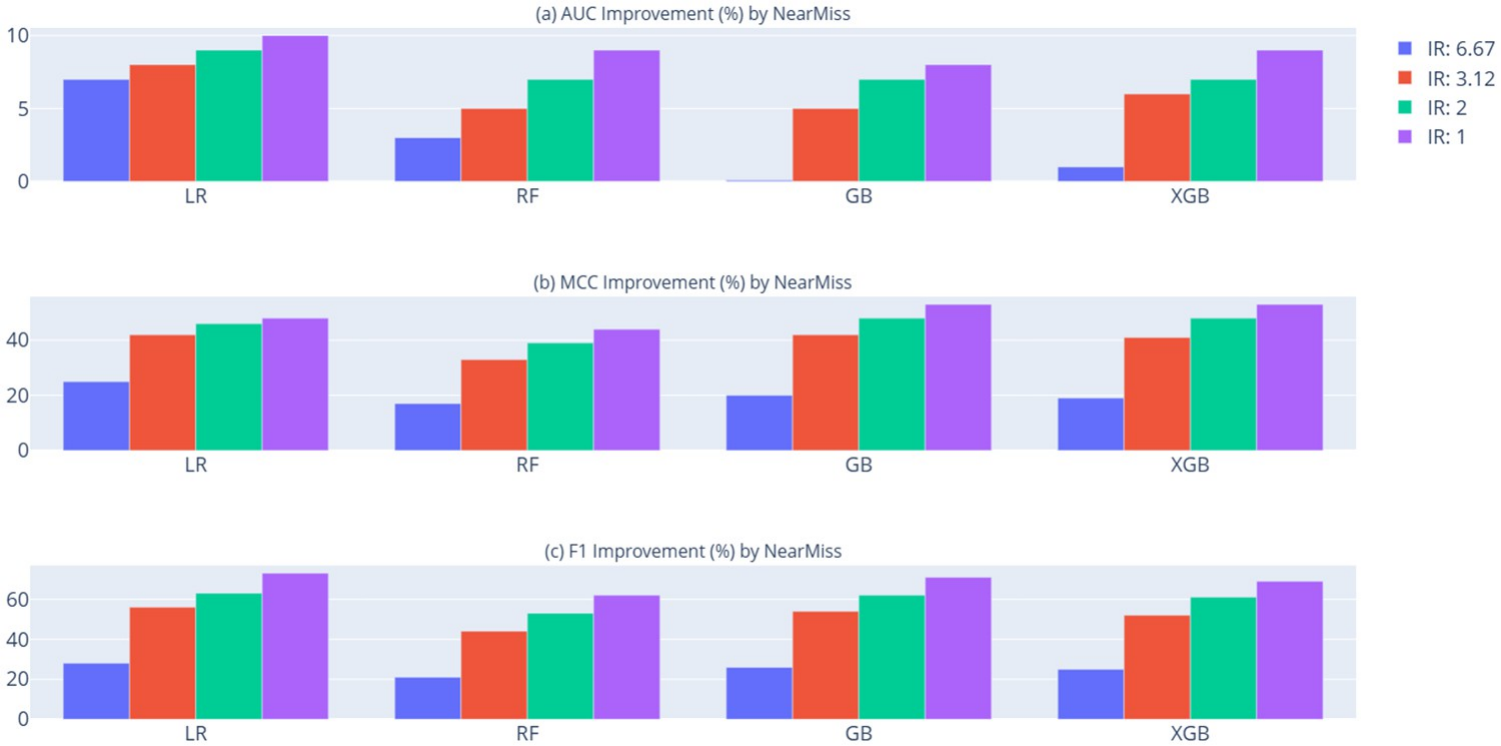
Performance enhancements of classification models using the undersampling method (NearMiss). (a) AUC improvement, (b) MCC improvement, and (c) F1 score improvement achieved by applying NearMiss in comparison to the original GMSC dataset across each model.

**Fig 7:**
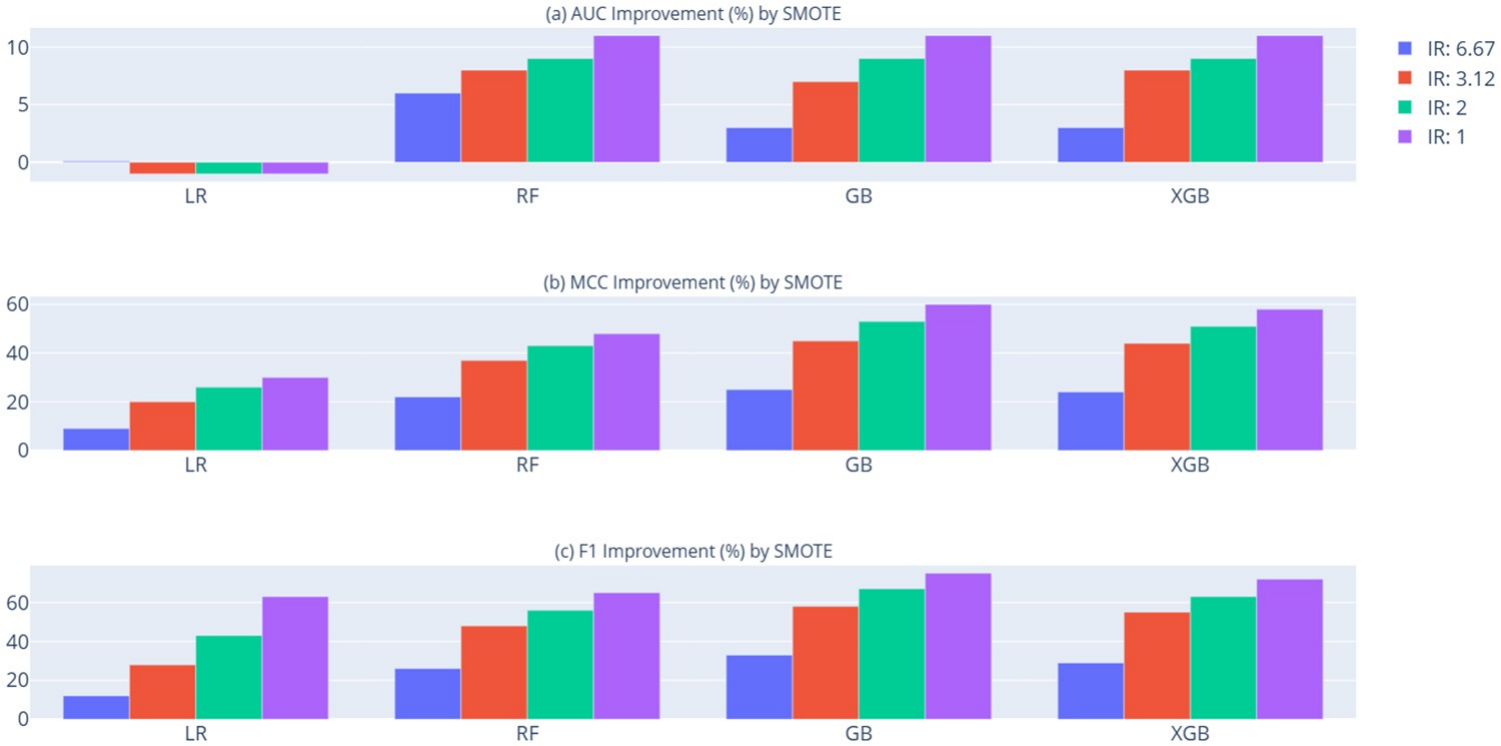
Performance enhancements of classification models using the oversampling method (SMOTE). (a) AUC improvement, (b) MCC improvement, and (c) F1 score improvement achieved by applying SMOTE in comparison to the original GMSC dataset across each model.

Table 7 presents the AUC, MCC, and F1 score comparison of tree-based ensemble classifiers relative to Logistic Regression (LR) as a benchmark, across various Imbalance Ratio (IR) achieved through undersampling and oversampling techniques. Figs 6 and 7 illustrate the AUC, MCC, and F1 score improvements obtained using NearMiss and SMOTE, respectively. As shown in Table 7, Figs 6 and 7, most classifiers demonstrate improved performance across AUC, MCC, and F1 metrics at lower imbalance ratios when both NearMiss undersampling and SMOTE oversampling are applied. For Gradient Boosting (GB), while undersampling with NearMiss improves MCC and F1 scores as the Imbalance Ratio (IR) decreases, a slight reduction (0.0008) in AUC is observed when the IR is 6.67. Similarly, for logistic regression, while SMOTE oversampling improves MCC and F1 scores with decreasing IR, a slight decline of approximately 1% is observed in AUC performance. These results suggest that achieving more balanced class distributions tends to yield better predictive outcomes. Overall, the findings indicate that resampling methods effectively address imbalanced datasets in tree-based classification models, with SMOTE (an oversampling technique) providing more substantial performance improvements than NearMiss (an undersampling approach).

**Table 7:** Performance comparison of AUC, MCC, and F1 across different IR on GMSC dataset.

**Oversampling #bad (using SMOTE)**	**Model**	**Best AUC**	**Best MCC**	**Best F1**
7% (original, IR = 13.96)	LR	0.7716	0.0948	0.0539
7%	RF	0.8242	0.2229	0.2037
7%	GB	0.8542	0.2058	0.1579
7%	XGB	0.8515	0.2165	0.1827
7%	Bayes Net [47]	0.8550	N/A	N/A
13% (IR = 6.67)	LR	0.7731	0.1895	0.1776
13%	RF	0.8829	0.4386	0.4604
13%	GB	0.8819	0.4569	0.4907
13%	XGB	0.8863	0.4536	0.4769
24% (IR = 3.12)	LR	0.7629	0.2916	0.3342
24%	RF	0.9080	0.5969	0.6807
24%	GB	0.9272	0.6585	0.7338
24%	XGB	0.9275	0.6540	0.7302
33% (IR = 2)	LR	0.7596	0.3543	0.4830
33%	RF	0.9189	0.6534	0.7645
33%	GB	0.9464	0.7367	0.8232
33%	XGB	0.9439	0.7244	0.8148
50% (IR = 1)	LR	0.7591	0.3935	0.6860
50%	RF	0.9311	0.7033	0.8550
50%	GB	**0.9649**	**0.8104**	**0.9072**
50%	XGB	0.9611	0.7974	0.9007
**Undersampling #good (using NearMiss)**	**Model**	**Best AUC**	**Best MCC**	**Best F1**
7% (original, IR = 13.96)	LR	0.7716	0.0948	0.0539
7%	RF	0.8242	0.2229	0.2037
7%	GB	0.8542	0.2058	0.1579
7%	XGB	0.8515	0.2165	0.1827
7%	Bayes Net [47]	0.8550	N/A	N/A
13% (IR = 6.67)	LR	0.8407	0.3439	0.3381
13%	RF	0.8562	0.3978	0.4131
13%	GB	0.8534	0.4032	0.4203
13%	XGB	0.8575	0.4058	0.4313
24% (IR = 3.12)	LR	0.8532	0.5197	0.6147
24%	RF	0.8789	0.5573	0.6405
24%	GB	0.9015	0.6219	0.6953
24%	XGB	0.9067	0.6314	0.7017
33% (IR = 2)	LR	0.8586	0.5547	0.6864
33%	RF	0.8904	0.6163	0.7307
33%	GB	0.9199	0.6876	0.7822
33%	XGB	0.9253	0.7014	0.7918
50% (IR = 1)	LR	0.8683	0.5787	0.7822
50%	RF	0.9109	0.6631	0.8267
50%	GB	0.9383	0.7391	0.8669
50%	XGB	**0.9451**	**0.7506**	**0.8728**

Additionally, non-parametric models such as Random Forest (RF), Gradient Boosting (GB), and eXtreme Gradient Boosting (XGB) exhibit superior performance compared to Logistic Regression (LR) as the benchmark on both the original imbalanced dataset and the resampled balanced dataset, as demonstrated in Table 7. This finding suggests that non-parametric models possess a greater capacity to capture non-linear relationships in complex and non-linear datasets.

Furthermore, Engelmann and Lessmann (2021) [18] demonstrated that oversampling techniques in non-linear datasets yield the best classification performance when paired with tree-based models such as Random Forest (RF), Gradient Boosting (GB), and eXtreme Gradient Boosting (XGB). Consequently, the findings in Table 6 corroborate the results of Engelmann and Lessmann (2021) [18], as the GMSC dataset is a non-linear credit scoring dataset.

As illustrated in Table 7, Figs 6 and 7, Gradient Boosting (GB) consistently achieves the highest performance across all metrics—AUC, MCC, and F1 score—when applying SMOTE-based oversampling. On the other hand, eXtreme Gradient Boosting (XGB) exhibits superior performance across these metrics under NearMiss-based undersampling. These findings indicate that SMOTE-based oversampling provides the most effective class-balancing approach, yielding optimal classification outcomes on the GMSC dataset.

Based on the results in Table 8, our analysis indicates that Gradient Boosting (GB) combined with SMOTE oversampling emerges as the top-performing model, demonstrating the highest AUC, MCC, and F1 scores among all evaluated methods. Specifically, the GB model with SMOTE achieved an AUC of 0.9649, an MCC of 0.8104, and an F1 score of 0.9072. Furthermore, the GB model with SMOTE surpasses the recent benchmark performance [18] attained through GB with conditional Wasserstein Generative Adversarial Network (cWGAN)-based oversampling. This benchmark analysis, combined with previous findings in the credit scoring literature [18], provides a foundation for evaluating NATE’s performance against established standards. Specifically, it assesses NATE’s ability to enhance classification outcomes in non-linear and imbalanced datasets, highlighting its comparative effectiveness within the context of prior research in the domain. This high level of predictive accuracy, however, is accompanied by substantial computational costs, with processing times significantly longer than those of the XGBoost model paired with NearMiss undersampling. All computations were conducted on an Intel Xeon Gold 6246R CPU @ 3.40 GHz, utilizing 32 cores.

**Table 8:** Evaluation of benchmark and optimal model performance with resampling techniques. Benchmark based on cWGAN oversampling [18].

Metrics	Engelmann and Lessmann [18]	GB using SMOTE	XGB using NearMiss
AUC	0.8304	0.9649 (2257.23 seconds)	0.9451 (35.27 seconds)
MCC	N/A	0.8104 (2265.41 seconds)	0.7506 (28.96 seconds)
F1	N/A	0.9072 (2265.69 seconds)	0.8728 (29.65 seconds)

*cWGAN: conditional Wasserstein Generative Adversarial Networks.

In comparison, while the XGB model with NearMiss undersampling achieved relatively competitive results, particularly with reduced processing times (e.g., 35.27 seconds for AUC), it fell short in terms of accuracy metrics. This indicates a trade-off between computational efficiency and predictive performance. The substantial time requirements associated with the GB model suggest it is ideal for applications where accuracy is the primary concern, whereas XGB with NearMiss serves as a feasible alternative in scenarios where computational resources or real-time processing is prioritized.

In conclusion, these findings highlight the robustness of the GB model with SMOTE in handling imbalanced datasets. However, practical applications of this model should carefully weigh computational constraints, especially in real-world contexts where processing efficiency is critical. Future research should focus on enhancing the computational efficiency of these models to achieve a balanced solution that effectively reconciles both accuracy and processing time.

### Performance comparison between oversampling and undersampling

The performance of classifiers using undersampling and oversampling techniques is compared at equivalent imbalance ratios to assess the impact of these resampling methods. Differences in AUC, MCC, and F1 scores are calculated by subtracting the metrics achieved through undersampling from those achieved through oversampling, using identical imbalance ratios for the resampled datasets across different sampling methods.

As demonstrated in Table 9, most classifiers exhibit improved performance with the application of oversampling techniques, reflected in positive increases in AUC, MCC, and F1 scores, with the exception of Logistic Regression (LR). For instance, the AUC for the Random Forest (RF) model increases by 0.0267, 0.0291, 0.0285, and 0.0202 when the proportion of bad credit samples reaches 13%, 24%, 33%, and 50% of the dataset, respectively. Similarly, the MCC for Gradient Boosting (GB) improves by 0.0537, 0.0366, 0.0491, and 0.0713 under these same conditions. In addition, the F1 score for eXtreme Gradient Boosting (XGB) rises by 0.0456, 0.0285, 0.0230, and 0.0279 across these increasing proportions of bad credit samples. These findings highlight the effectiveness of oversampling techniques in significantly enhancing the classification capabilities of non-parametric, tree-based models in handling imbalanced datasets.

**Table 9:** Increase in AUC, MCC, and F1 between oversampling and undersampling.

% of Minority	Model	AUC (over-under)	MCC (over-under)	F1 (over-under)
13% (IR = 6.67)	LR	-0.0676	-0.1544	-0.1605
13%	RF	0.0267	0.0408	0.0473
13%	GB	0.0285	0.0537	0.0704
13%	XGB	0.0288	0.0478	0.0456
24% (IR = 3.12)	LR	-0.0903	-0.2281	-0.2805
24%	RF	0.0291	0.0396	0.0402
24%	GB	0.0257	0.0366	0.0385
24%	XGB	0.0208	0.0226	0.0285
33% (IR = 2)	LR	-0.0990	-0.2004	-0.2034
33%	RF	0.0285	0.0371	0.0338
33%	GB	0.0265	0.0491	0.0410
33%	XGB	0.0186	0.0230	0.0230
50% (IR = 1)	LR	-0.1092	-0.1852	-0.0962
50%	RF	0.0202	0.0402	0.0283
50%	GB	0.0266	0.0713	0.0403
50%	XGB	0.0160	0.0468	0.0279

### Interpretability

**Fig 8:**
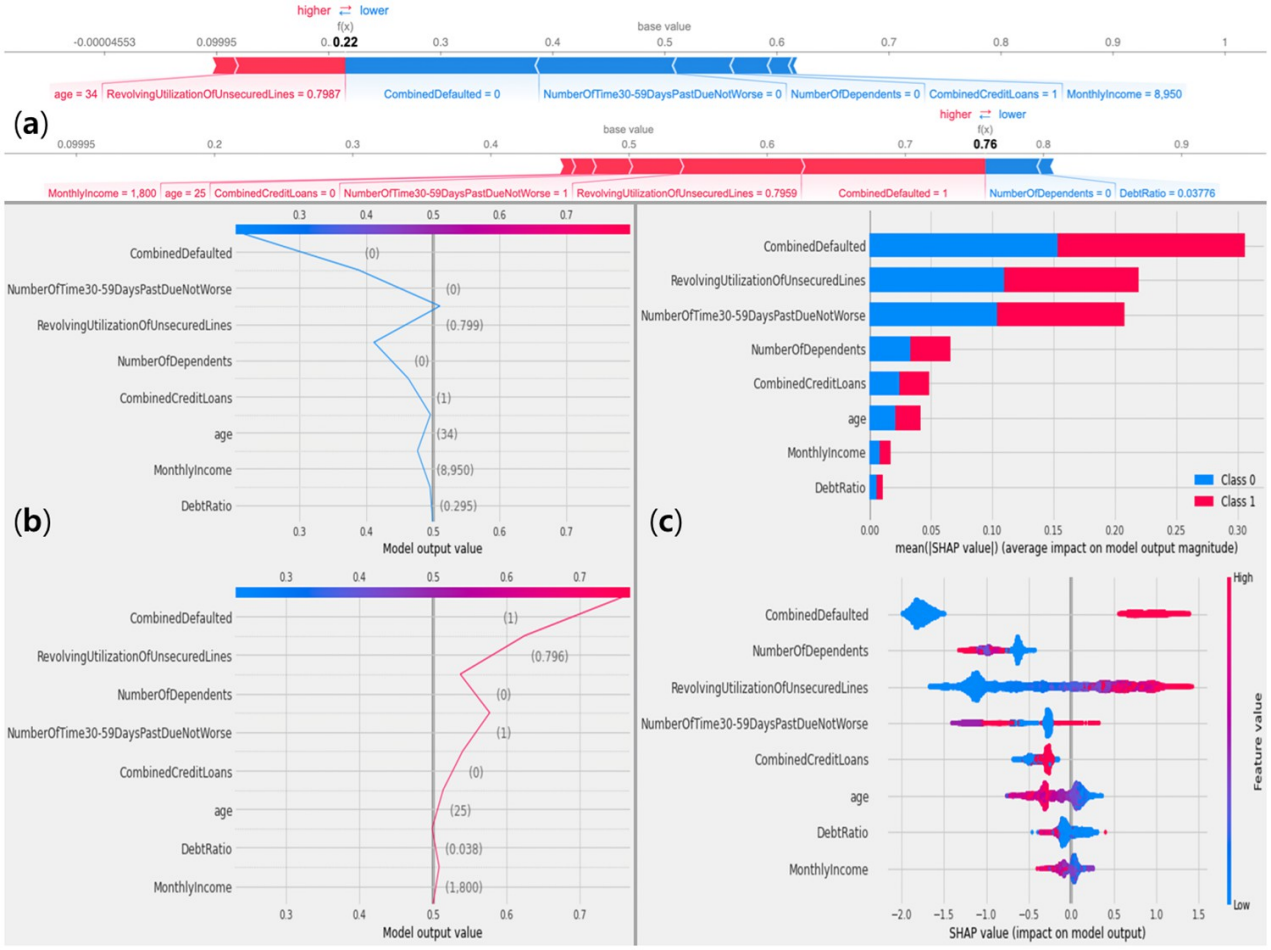
Local and global interpretability of NATE. A SHAP analysis for credit default prediction on GMSC dataset (a) force plots, (b) decision plots, and (c) bar plot (top) and violin plot (bottom).

The interpretability plots presented in Fig 8 offer a comprehensive analysis of the model’s decision-making process by integrating both local and global interpretability methods, thus facilitating a more refined understanding of individual predictions and overall feature importance.

Fig 8(a) presents force plots that offer local explanations for two specific instances, illustrating the contribution of each feature to the model’s prediction. In Fig 8(a), the prediction is 0.22, which falls below the 0.5 threshold, indicating a classification of non-default. Features such as ‘age (34)’ and ‘RevolvingUtilizationOfUnsecuredLines (0.7987)’ contribute to shifting the prediction toward default, while factors like ‘CombinedDefaulted (0)’ and ‘NumberOfTimes30-59DaysPastDueNotWorse (0)’ lower the prediction, resulting in a non-default classification. In Fig 8(a), the prediction is 0.76, which is above the 0.5 threshold, signifying a default classification. Key features contributing to this outcome include ‘CombinedDefaulted (1)’ and ‘RevolvingUtilizationOfUnsecuredLines (0.7956)’, with additional factors like ‘NumberOfDependents (0)’ and ‘DebtRatio (0.03776)’ slightly pulling the prediction toward non-default.

Fig 8(b) presents decision plots that provide insights into how cumulative feature contributions affect model outputs for different classes. In the decision plot as shown in Fig 8(b), which focuses on a non-default instance, features such as ‘CombinedDefaulted (0)’ and ‘NumberOfTimes30-59DaysPastDueNotWorse (0)’ significantly decrease the predicted probability of default. The upward slope of the blue lines demonstrates how these features guide the prediction toward non-default. Conversely, the decision plot as shown in Fig 8(b) highlights a default instance, where features like ‘RevolvingUtilizationOfUnsecuredLines (0.796)’ and ‘CombinedDefaulted (1)’ raise the predicted probability, pushing the red lines higher as the decision trends toward default.

Fig 8(c) illustrates global feature importance. Fig 8(c)’s bar plot shows the average global importance of features across all instances, with ‘CombinedDefaulted’ having the highest mean SHAP value, indicating its substantial influence on model predictions. Other critical features include ‘RevolvingUtilizationOfUnsecuredLines’, ‘NumberOfTimes30-59DaysPastDueNotWorse’, all of which significantly impact the likelihood of default. Fig 8(c)’s violin plot provides a detailed view of SHAP value distributions for each feature, showing the extent to which each feature affects predictions across instances. Wider sections for features like ‘CombinedDefaulted’ and ‘RevolvingUtilizationOfUnsecuredLines’ indicate considerable variation in their contributions across the dataset.

These SHAP plots offer a layered approach to model interpretability, effectively balancing local and global explanations. At the local level, the force and decision plots allow users to analyze individual predictions, while at the global level, the bar and violin plots reveal the most influential features across the entire dataset. This integrated analysis is especially valuable in high-stakes applications such as credit risk assessment, where understanding both individual predictions and overarching trends is crucial to fostering a transparent and trustworthy AI system. By considering both specific cases and general patterns, these visualizations provide comprehensive insights into model behavior, thereby enhancing the interpretability and accountability of the machine learning process.

This approach aligns with recent research advocating for a shift toward hypothesis-driven decision-making in explainable AI [48]. The integration of both local and global insights is consistent with the evaluative AI framework suggested in these studies, in which decision-makers actively engage with the AI system to generate and test hypotheses, rather than passively receiving recommendations [48]. By examining both supporting and opposing evidence for specific decisions, evaluative AI reduces the risk of over-reliance on automated recommendations and promotes a deeper understanding of decision-making processes. The SHAP plots embody this approach by enabling users to explore both localized and overarching explanations, thereby fostering evidence-based engagement with the model’s reasoning.

## Discussion

### Highlights and limitations

To address class imbalance, re-sampling techniques are frequently utilized to balance class distributions by either reducing instances from the majority class or augmenting those from the minority class. Among these, the Synthetic Minority Over-sampling TEchnique (SMOTE) is widely used, as it generates synthetic samples for the minority class by leveraging the available data itself [30]. While oversampling methods like SMOTE fully utilize existing data, undersampling techniques, such as NearMiss, balance the dataset by discarding a portion of majority class instances [12]. Oversampling is generally preferred over undersampling, as it preserves complete data information, often resulting in improved performance for machine learning models trained on larger datasets.

Despite these advantages, integrating SMOTE within the NATE framework presents certain limitations. While SMOTE effectively improves minority class representation, it can also introduce overlapping data points, which may be perceived as additional noise, potentially leading to overfitting [18]. This limitation is particularly relevant for NATE, given its non-parametric, tree-based structure, which is inherently flexible but also susceptible to overfitting without careful tuning.

Furthermore, while the results obtained from the Give Me Some Credit (GMSC) dataset are encouraging, we acknowledge that restricting the evaluation of the NATE framework to a single dataset represents a limitation of this study. This limitation has been addressed by emphasizing the need for further validation across a broader range of credit scoring datasets to ensure greater generalizability.

### Future work

A promising direction for future research involves investigating methods within the NATE framework to address overfitting risks associated with SMOTE while also improving the computational efficiency of NATE. This would aim to achieve a balanced solution that optimally integrates both predictive accuracy and processing efficiency. Furthermore, integrating Generative Adversarial Networks (GANs) or ADS-GAN (Anonymization through Data Synthesis using GANs) within the NATE framework may offer an effective approach for managing class imbalance. These methods can generate synthetic data closely aligned with the original distribution, potentially providing a more realistic and diverse oversampling solution [2]. Such integration could enhance the NATE framework’s ability to handle class imbalance effectively while preserving generalization capabilities, thereby addressing current limitations and advancing the quality of synthetic data generation for imbalanced datasets.

Beyond the integration of generative models to synthesize minority class samples, we also recognize the importance of validating the generalizability of the NATE framework further. Accordingly, future research will involve extending the evaluation to additional publicly available credit scoring datasets, including the German, Australian, Taiwanese, and Polish datasets from the UCI Machine Learning Repository. This broader evaluation will enable a more comprehensive assessment of the framework’s robustness and applicability across diverse contexts. By incorporating a wider range of datasets, we aim to deepen our understanding of NATE’s effectiveness and ensure its adaptability to various credit scoring scenarios.

## Conclusion

In this study, a non-parametric approach for explainable credit scoring using XAI techniques was proposed, examining varying class distributions within the GMSC dataset. The research demonstrated the robustness of non-parametric models when compared to the parametric Logistic Regression (LR) model, which is commonly employed as the standard in the field of credit scoring. Moreover, the study highlighted the effectiveness of resampling techniques in addressing substantial class imbalances. The classification performance was evaluated using AUC, MCC, and F1 score metrics.

The experimental results indicated that non-parametric tree-based ensemble models, particularly Gradient Boosting (GB) and eXtreme Gradient Boosting (XGB), outperformed Logistic Regression (LR) on both balanced datasets and the original, significantly imbalanced dataset. The classification performance of tree-based ensemble models improved as the dataset’s imbalance ratio decreased, indicating that a lower imbalance ratio corresponds to better performance. In the comparison of resampling techniques, SMOTE demonstrated superior results compared to NearMiss on the imbalanced dataset. The effectiveness of the SMOTE oversampling method in non-parametric tree-based models was consistent across all imbalance ratios investigated in this study.

To address the limitations of Logistic Regression (LR) and balance the trade-off between accuracy and interpretability, a non-parametric tree-based model paired with TreeExplainer was utilized to enhance classification performance and provide both local and global model interpretation. Individual predictions, as well as overall model predictions, were analyzed based on the contribution of features through SHAP values. Consequently, the proposed NATE (Non-pArameTric approach for Explainable credit scoring) as an XAI method facilitates the creation of an explainable credit scoring model suitable for practical applications. This approach allows for the comprehensive evaluation and explanation of risk factors, while maintaining high predictive accuracy for credit applicants.
